# Pancreatic Exocrine Insufficiency and Adenovirus—Is There a Link?

**DOI:** 10.1002/ueg2.12771

**Published:** 2025-02-10

**Authors:** Christoph Ammer‐Herrmenau, Evgenii Shumilov

**Affiliations:** ^1^ Department of Gastroenterology, Gastrointestinal Oncology and Endocrinology University Medical Center Goettingen Gottingen Germany; ^2^ Department of Medicine A for Hematology, Oncology and Pneumology University Hospital Muenster Muenster Germany

Diarrhoea is a highly prevalent symptom following haematopoietic stem cell transplantation (HSCT) with several differential diagnosis: graft‐versus‐host disease, infections (*Clostridioides difficile*, *CMV*, *Cryptosporidium* sp.), toxicity of chemotherapy, damage due to radiation, necrotizing enterocolitis, or transplant associated microangiopathy [1, 2].

In this issue, Launspach et al. conducted a prospective and longitudinal trial to investigate the incidence and impact of pancreatic exocrine insufficiency (PEI) in children and young adults after HSCT [3]. Starting prior HSCT until 1‐year post‐HSCT pancreatic elastase (PE) was longitudinally analysed in 83 patients. Importantly, patients with known pancreatic diseases were excluded. The proportion of patients with decreased level of PE < 200 µg/g peaked 1‐month post‐HSCT (42%) including samples with liquid consistency. When only solid stool samples were considered, the prevalence of reduced PE remained high with 21%. Transabdominal ultrasound of the pancreas revealed that almost 80% of patients with decreased PE had noticeable imaging findings with increased echogenicity, irregular organ texture or signs of pancreas atrophy. Notably, patients with reduced PE for more than 4 weeks experienced a significant decrease of body mass index (BMI) post‐HSCT compared to patients with normal PE suggesting a clinically relevant malabsorption syndrome. In addition, patients with reduced PE showed a higher risk of non‐relapse mortality (hazard ratio, HR 4.9) in a 3‐year survival analysis, along with a prolonged initial HSCT hospitalization. Furthermore, the authors observed more severe adverse events during anti‐cancer treatment and an increased rate of erythrocytes and platelets transfusions compared to patients with normal PE. Finally, multivariable analysis revealed adenovirus infection (HR 3.5) and higher pre‐HSCT body mass index (BMI, HR 1.38) as significant independent factors for reduced PE.

Although Launspach et al. [3] did not conduct a combined assessment of functional tests, symptoms and nutritional status as suggested by the recently published European guidelines for the diagnosis and treatment of pancreatic exocrine insufficiency [4], the subgroup of patients with reduced PE revealed a significant decline in BMI indicating a potential malabsorption.

Even if some individuals received pancreatic enzyme replacement therapy (PERT) this therapeutic intervention was not systematically assessed. PERT is considered a well‐tolerated therapeutic approach improving nutritional status and quality of life in patients with PEI [5, 6]. Thus, PERT should be considered in post HSCT patients more often and prospective clinical trials should investigate the clinical benefits of it.

The impaired functional exocrine capacity of the pancreas that was associated with structural alterations on imaging might be multifactorial and caused by toxicity of chemotherapy, radiation or fasting. However, the authors strikingly observed an association of adenovirus infections and reduced PE in their study population suggesting a potential, yet unexplored functional link between this virus and the exocrine function. Case series have reported acute pancreatitis caused by adenovirus infection in immunosuppressed individuals, including children following HSCT [7]. However, no acute pancreatitis cases was reported in this cohort.

In summary, Launspach and colleagues identified an unexpectedly high incidence of reduced PE in children and young adults after HSCT, which was associated with increased mortality and morbidity and was linked to higher BMI before HSCT and adenovirus infections [3]. If confirmed in prospective trials, these novel findings might have direct clinical implications regarding routine testing of pancreatic exocrine function and subsequent PERT in post HSCT patients. It will also be interesting to see whether these results can be recapitulated in adult patients and whether there is indeed a functional link between adenovirus infections and PEI.

## Conflicts of Interest

The authors declare no conflicts of interest.

## Data Availability

The authors have nothing to report.

## References

[1] K. Robak , J. Zambonelli , J. Bilinski , and G. W. Basak , “Diarrhea After Allogeneic Stem Cell Transplantation: Beyond Graft‐Versus‐Host Disease,” European Journal of Gastroenterology and Hepatology 29, no. 5 (2017): 495–502, 10.1097/meg.0000000000000833. 28067684

[2] H. H. Tuncer , N. Rana , C. Milani , et al., “Gastrointestinal and Hepatic Complications of Hematopoietic Stem Cell Transplantation,” World Journal of Gastroenterology 18 (2012): 1851–1860. 22563164 10.3748/wjg.v18.i16.1851 PMC3337559

[3] M. Launspach , A. Mindermann , J. Schulz , et al., “High Incidence and Impact of Suspected Exocrine Pancreatic Insufficiency in Patients Post-Hematopoietic Stem Cell Transplantation: A Single-Center Prospective Observational Study,” United European Gastroenterology Journal (forthcoming), 10.1002/ueg2.12769. PMC11975613 39955611

[4] J. E. Dominguez‐Muñoz , M. Vujasinovic , D. de la Iglesia , et al., “European Guidelines for the Diagnosis and Treatment of Pancreatic Exocrine Insufficiency: UEG, EPC, EDS, ESPEN, ESPGHAN, ESDO, and ESPCG Evidence‐Based Recommendations,” United European Gastroenterology Journal (2024). 10.1002/ueg2.12674 PMC11866322 39639485

[5] J. G. D'Haese , G. O. Ceyhan , I. E. Demir , et al., “Pancreatic Enzyme Replacement Therapy in Patients With Exocrine Pancreatic Insufficiency Due to Chronic Pancreatitis: A 1‐Year Disease Management Study on Symptom Control and Quality of Life,” Pancreas 43, no. 6 (2014): 834–841, 10.1097/mpa.0000000000000131. 24717829

[6] N. Gubergrits , E. Malecka‐Panas , G. A. Lehman , et al., “A 6‐Month, Open‐Label Clinical Trial of Pancrelipase Delayed‐Release Capsules (Creon) in Patients With Exocrine Pancreatic Insufficiency Due to Chronic Pancreatitis or Pancreatic Surgery,” Alimentary Pharmacology & Therapeutics 33, no. 10 (2011): 1152–1161, 10.1111/j.1365-2036.2011.04631.x. 21418260

[7] C. M. Bateman , A. M. Kesson , and P. J. Shaw , “Pancreatitis and Adenoviral Infection in Children After Blood and Marrow Transplantation,” Bone Marrow Transplantation 38, no. 12 (2006): 807–811, 10.1038/sj.bmt.1705526. 17057728

